# Loss of epidermal AP1 transcription factor function reduces filaggrin level, alters chemokine expression and produces an ichthyosis-related phenotype

**DOI:** 10.1038/cddis.2017.238

**Published:** 2017-06-01

**Authors:** Christina A Young, Ellen A Rorke, Gautam Adhikary, Wen Xu, Richard L Eckert

**Affiliations:** 1 Departments of Biochemistry and Molecular Biology, University of Maryland School of Medicine, Baltimore, MD 21201, USA; 2 Departments of Microbiology and Immunology, University of Maryland School of Medicine, Baltimore, MD 21201, USA; 3 Departments of Dermatology, University of Maryland School of Medicine, Baltimore, MD 21201, USA; 4 Departments of Reproductive Biology, University of Maryland School of Medicine, Baltimore, MD 21201, USA; 5 Marlene and Stewart Greenebaum Comprehensive Cancer, University of Maryland School of Medicine, Baltimore, MD 21201, USA

## Abstract

AP1 transcription factors are important controllers of epidermal differentiation. Multiple family members are expressed in the epidermis in a differentiation-dependent manner, where they function to regulate gene expression. To study the role of AP1 factor signaling, TAM67 (dominant-negative c-jun) was inducibly expressed in the suprabasal epidermis. The TAM67-positive epidermis displays keratinocyte hyperproliferation, hyperkeratosis and parakeratosis, delayed differentiation, extensive subdermal vasodilation, nuclear loricrin localization, tail and digit pseudoainhum and reduced filaggrin level. These changes are associated with increased levels of IFN*γ*, CCL3, CCL5, CXCL9, CXCL10, and CXCL11 (Th1-associated chemokines), and CCL1, CCL2, CCL5 and CCL11 (Th2-associated chemokines) in the epidermis and serum. S100A8 and S100A9 protein levels are also markedly elevated. These changes in epidermal chemokine level are associated with increased levels of the corresponding chemokine mRNA. The largest increases were observed for CXCL9, CXCL10, CXCL11, and S100A8 and S100A9. To assess the role of CXCL9, CXCL10, CXCL11, which bind to CXCR3, on phenotype development, we expressed TAM67 in CXCR3 knockout mice. Using a similar strategy, we examine the role of S100A8 and S100A9. Surprisingly, loss of CXCR3 or S100A8/A9 did not attenuate phenotype development. These studies suggest that interfering with epidermal AP1 factor signaling initiates a loss of barrier function leading to enhanced epidermal chemokine production, but that CXCR3 and S100A8/A9 do not mediate the phenotypic response.

The epidermis produces a barrier that functions to prevent loss of body fluids, fights infection, and is essential for life. Epidermal keratinocytes differentiate to form the barrier via a process that involves systematic destruction of intracellular structures leaving a network of covalently-crosslinked proteins, lipids, and keratin bundles that form the barrier.^1^ AP1 transcription factors are essential regulators of this process.^2, 3, 4, 5, 6, 7^ They form homo- and heterodimers that bind DNA response elements to regulate gene expression.^8^ An example is the AP1 site in the involucrin gene enhancer that is required for expression of involucrin in epidermis.^2, 3, 4, 5, 6, 7^ Disruption of AP1 factor function can produce mouse phenotypes that mimic human epidermal diseases.^9, 10, 11, 12^ To investigate the role of AP1 factors in epidermis, we inactivated AP1 transcription factor function in the suprabasal epidermis using TAM67, a dominant-negative form of c-jun.^13, 14, 15^ TAM67 dimerizes with AP1 factor family members, but the resulting complex is not able to activate transcription. These animals display an epidermal phenotype including increased cell proliferation and delayed differentiation, extensive epidermal hyperkeratosis and parakeratosis, nuclear loricrin accumulation and digit and tail autoamputation.^9^

Epidermal ultrastructure studies reveal reduced cornified envelope thickness, and abnormal desmosome, keratin filament and lamellar body morphology.^16^ Analysis of cornified envelope composition reveals reduced levels of cutaneous keratins, late envelope precursor proteins, hair-related proteins, and increased levels of hyperproliferation-associated keratins and proline-rich proteins.^16^ Moreover, changes in protein level were paralleled by changes in messenger RNA (mRNA) level. Thus, suprabasal AP1 factor inactivation reduces expression of AP1 factor-responsive late differentiation genes and increases expression of early differentiation genes. An important change is a reduction in expression of filaggrin family-related genes,^16^ a change that is frequently observed in ichthyosis vulgaris and atopic dermatitis.^17, 18, 19, 20^ In these diseases, compromised barrier function is associated with migration of immune cells into the epidermis.^21^ These immune cells are thought to contribute to the development of disease phenotype due to the stimulatory impact of chemokines and cytokines on keratinocyte proliferation.^21^ In the present study, we characterize biochemical, structural and immune changes following inhibition of AP1 factor function in the suprabasal epidermis. We show that structural proteins are altered in level, barrier function is compromised, and IFN*γ*, CCL3, CCL5, CXCL9, CXCL10 and CXCL11 (Th1-associated), and CCL1, CCL2, CCL5 and CCL11 (Th2-associated) chemokines are elevated. S100A8 and S100A9 levels are also increased. To begin to understand the role of selected chemokines in phenotype development, we show that the CXCR3 receptor and it ligands (CXCL9, CXCL10, CXCL11) and S100A8 and S100A9, although markedly overexpressed in TAM67-positive epidermis, are not required for phenotype development.

## Results

**Figure 1:**
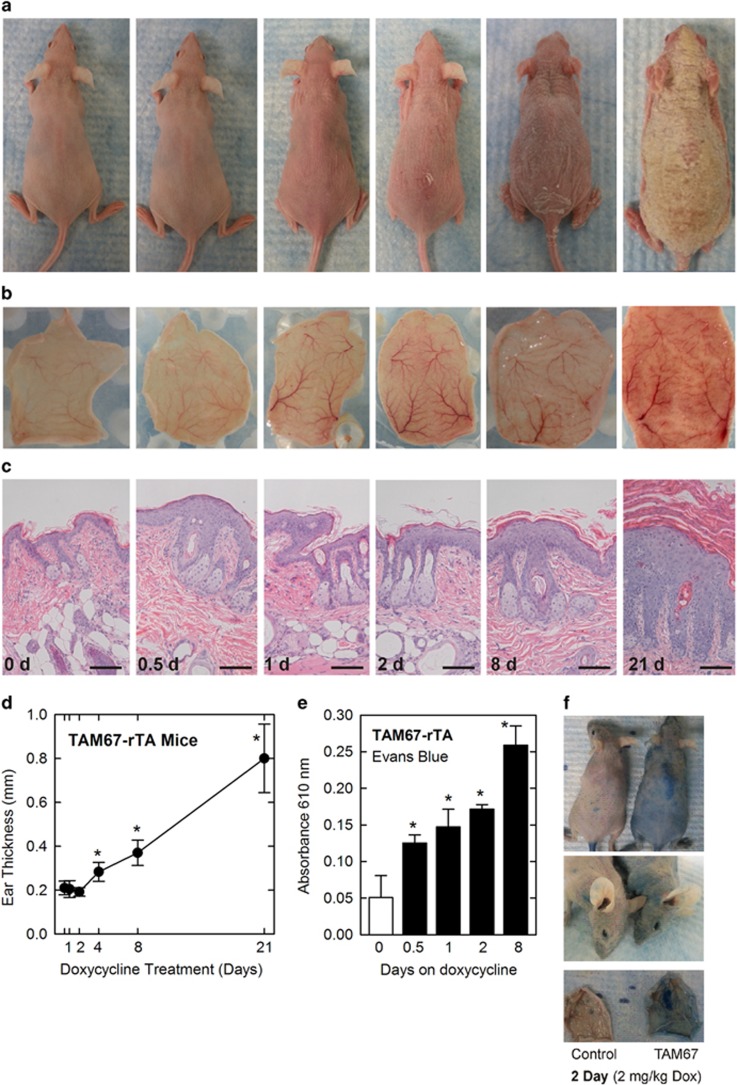
Development of TAM67 phenotype TAM67-rTA mice treated with 2 mg/ml doxycycline in drinking water for 0–21 d. (**a**) TAM67-rTA mice were photographed at 0, 0.5, 1, 2, 8, and 21 d following initiation of doxycycline treatment. (**b**) The back skin of TAM67-rTA mice was removed and flipped to reveal hypodermis and vascular network changes during phenotype development. (**c**) H&E stained sections of TAM67-rTA mouse skin. The bars=100 microns. (**d**) Measurement of TAM67-rTA mouse ear thickness at indicated times after initiation of doxycycline treatment. The values are mean±S.E.M., *n*=6, *P*<0.001. (**e,f**) The Evans Blue dye assay reveals enhanced vessel permeability. TAM67-rTA mice were treated with 2 mg/ml doxycycline for 0.5–8 d (three mice/group, black bars) and then injected with Evans Blue dye solution. The open bar indicates dye level in control (no doxycycline) mice. The values are mean±S.E.M. The treatment groups were significantly elevated compared to control (*n*=3, *P*<0.05). Photographs of day two mice indicate enhanced dye permeation into the epidermis. The bottom panel shows full thickness skin samples that were harvested following Evans Blue assay and are pictured inside up

We recently showed that inhibition of AP1 factor function in the suprabasal epidermis markedly changes the epidermal phenotype leading to an ichthyosis/keratoderma-like phenotype.^9, 16^ The present study expands upon these observations. Figure 1 shows the TAM67-FLAG time-dependent change in epidermal phenotype. Erythema is observed at 12–24 h after induction of TAM67 expression and extended TAM67 expression leads to formation of a thick scaly cornified coat at 21 d (Figure 1a). The phenotype is associated with increased blood flow to the skin, first observed at 12 and 24 h (Figure 1b). Histologic sections reveal an increase in epidermal thickness which is also apparent at 12–24 h, with a massive increase in epidermis and stratum corneum thickness observed at 21 d (Figure 1c). Ear thickness is also increased progressively and is four-fold thicker at 21 d (Figure 1d). The images in Figure 1b suggest a rapid change in blood flow to the skin that is evident at 24 h. To assess whether this is associated with increased vascular permeability, we injected Evans Blue dye and monitored its leakage from vessels into the surrounding tissue.^22^ Increased tissue Evans Blue dye accumulation is evident at 0.5 d and increases with time (Figure 1e). Epidermal dye accumulation is readily visible in the skin at 2 d after induction of TAM67 expression (Figure 1f), which indicates enhanced vessel permeability.

### Chemokine levels in serum and epidermis

**Figure 2:**
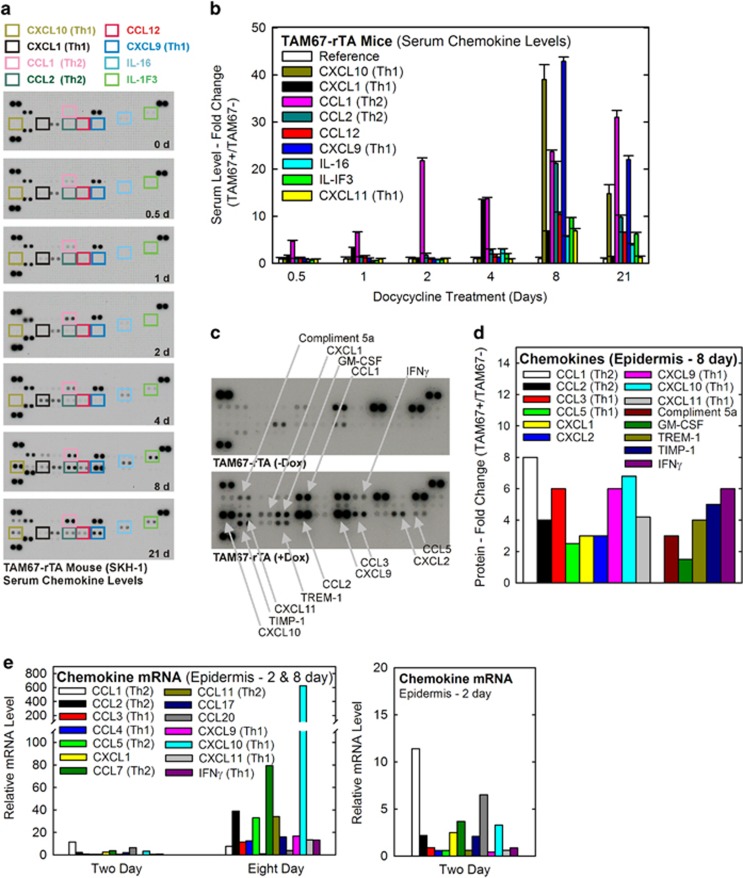
Chemokine levels in epidermis and serum (**a**) TAM67-rTA mice were treated with 2 mg/ml doxycycline in drinking water for 0–21 days and serum was analyzed for chemokine content. The boxes indicate chemokines that change in level. Similar changes were observed in each of three independent experimental replicates. Control dots (in duplicate) are at the lower and upper left, and the upper right. (**b**) Pixel density graph depicts the levels of selected cytokines at 0.5–21 d after initiation of doxycycline treatment. The values are normalized to the day 0 time point (not shown) and show fold change. The values are mean±S.E.M., *n*=3, *P*<0.005. Control dots (in duplicate) are at the lower and upper left, and the upper right in each panel. (**c**) TAM67-rTA mice were treated with or without doxycycline for 8 d and epidermal extracts were prepared and analyzed for chemokine content using the chemokine protein array. Upregulated chemokines are indicated. (**d**) The graph presents a pixel density plot of data derived from panel (**c**). Since the plot is a scan of a single experiment, as a representative example of three replicate experiments, error bars are not included. (**e**) RNA, extracted from epidermis on 0, 2, and 8 d after initiation of doxycycline treatment of TAM67-rTA mice, was reverse transcribed to cDNA and applied to the cytokine and chemokine RNA array. Data were analyzed by the comparative C_T_ method and plotted as relative mRNA level. The values are mean±S.E.M., *n*=3, *P*<0.005. The right panel shows the two day data on an expanded scale. The key for treatment groups in this panel is the same as in the left panel. Similar trends were observed in three replicate experiments. The data indicates fold change as compared to the control (day zero) sample

To identify events that contribute to this phenotype, we monitored serum chemokine content and observed elevated levels of a host of chemokines including CXCL1, CXCL10, CCL1, CCL2, CCL12, CXCL9, IL-16 and IL-1F3 (Figure 2a). Plotting chemokine level *versus* time reveals an early increase in CCL1 at 0.5 d with peak levels observed at days 2–21 (Figure 2b). CXCL1 level is elevated at 4 and 8 d, and at 21 d other chemokines, including CXCL10, CCL1, CCL2, CCL12, CXCL9, IL-16 and IL-IF3 are increased (Figure 2b). These findings indicate a strong early increase in CCL1 (0.5 d),^23^ followed by an increase in CCL2 and CXCL10, CXCL1, CXCL9, and CXCL11 chemokines at 8 and 21 days.

Chemokine levels are also altered in the epidermis. TAM67-rTA mice were treated for 8 d with doxycycline and then epidermal chemokine levels were measured. Figures 2c shows that CCL1 and CCL2, CCL3, CCL5, CXCL9, CXCL10, CXCL11, and IFN*γ* chemokines are elevated. CXCL1, CXCL2, GM-CSF, TREM-1, TIMP-1, and compliment 5 A levels are also increased (Figures 2c). To understand the mechanism of this regulation, we measured mRNA level for selected chemokines. Figure 2e shows an increase in mRNA encoding CCL1 (7.7-fold), CCL2 (39-fold), CCL5 (33-fold), CCL7 (79.3-fold), CCL11 (16.8-fold), CCL3 (11.5-fold), CCL4 (12.4-fold), CXCL9 (16.8-fold), CXCL10 (625-fold), CXCL11 (13.2-fold), and IFN*γ* (13.1-fold) at 8 d after TAM67 induction. CCL17 (16-fold) and CCL20 (4-fold) were also increased at 8 d. An expanded plot of chemokine mRNA changes at 2 d after doxycycline addition shows increased levels of CCL1 (11.4-fold), CCL2 (2.2-fold), CCL7 (3.6-fold), CXCL1 (2.5-fold), CCL17 (2.1-fold), CCL20 (6.5-fold), and CXCL10 (3.3-fold) (Figure 2e). These findings suggest that Th1- and Th2-associated chemokines are elevated in epidermis, and that these increases are associated with increased mRNA level.

### Immune cell invasion

**Figure 3:**
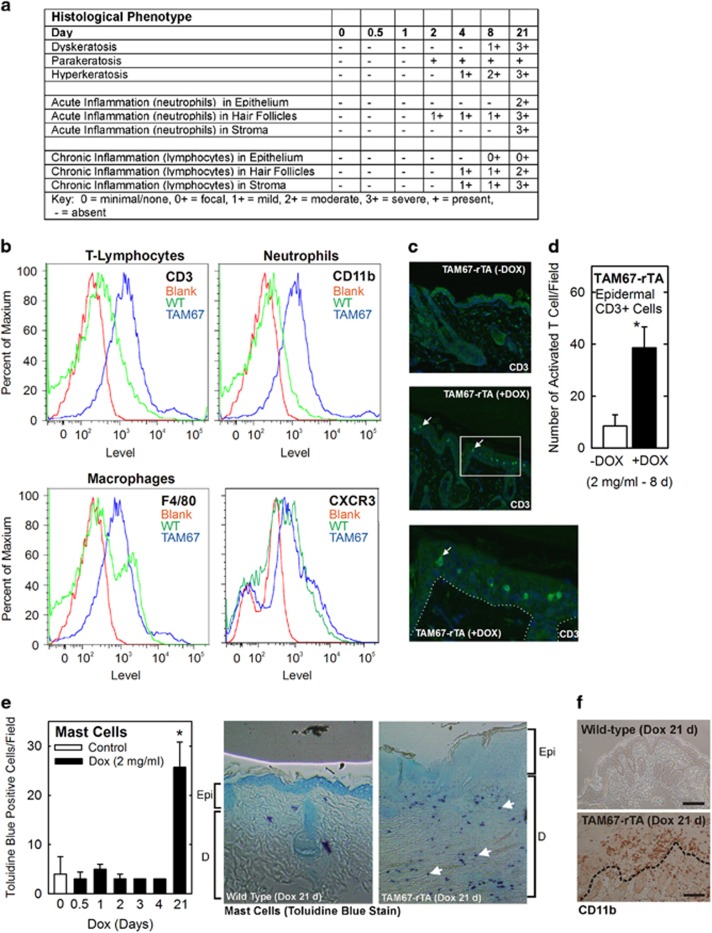
Immune cell involvement in TAM67-expressing skin (**a**) Epidermal/dermal sections were prepared from mice at the indicated time points and prevalence of the indicated parameters was monitored by counting. The findings were scored by an expert pathologist. (**b**) Flow cytometry data analysis of epidermal single cell suspensions from 8 d doxycycline treated WT and TAM67-rTA mice designed to detect CD3-, CD11b-, F4/80-, and CXCR3-positive cells. (Red-unstained/blank, green-WT, blue-TAM67-positive). CD11b-positive cells are labeled as neutrophils, but this population may also include dentritic cells. (**c,d**) Immunofluorescent staining for CD3 in TAM67-rTA mouse skin treated with or without doxycycline for 8 d. Bottom image is magnification of white box from middle image. The number of interfollicular CD3+ (activated T cells) cells per field in 11 separate images was counted and graphed (control – open box, doxycycline-treated – closed boxes). The arrows indicate CD3-positive cells in the suprabasal epidermis. The values are mean±S.E.M., *n*=11. The asterisk indicates a significant change, *P*<0.001. (**e**) Toluidine blue stain to detect dermal mast cells in TAM67-rTA mice treated with doxycycline for 0–21 d. (Epi, epidermis; D, dermis). The values are mean±S.E.M. and the asterisk indicates a significant change, *P*<0.001 (*n*=3). The arrows indicate mast cells in the dermis. (**f**) Detection of CD11b-positive cells in wild-type and TAM67-positive mice at 21 d after initiation of doxycycline treatment. The bar=100 μm and the dashed line separates the epidermis and dermis

We next monitored the types of immune cells present in the dermis/epidermis in response to epidermal TAM67 expression. Histologic examination of H&E-stained sections revealed the presence of parakeratosis beginning at 2 d, mild to moderate dyskeratosis and hyperkeratosis at 8 days and severe dyskeratosis/hyperkeratosis at 21 d (Figure 3a). Inflammation was detected in hair follicles, epidermis and stroma, and increased in severity with time. Consistent with these changes, cell sorting analysis reveals a marked increase in T-lymphocytes (CD3), neutrophils (CD11b), and macrophages (F4/80) (Figure 3b). We also assessed the number of CXCR3-positive cells (receptor for CXCL9, 10, 11) and found no change (Figure 3b). To identify the tissue localization of invading leukocytes, we stained epidermis from 8 d doxycycline-treated mice with antibodies specific for various immune cells. There was a four-fold enrichment of CD3-positive lymphocytes in the epidermal suprabasal layers (Figures 3c), and a substantial increase in mast cells (Figure 3e) and CD11b-positive neutrophils (Figure 3f) at 21 d.

### Role of CXCL9, CXCL10, CXCL11 in phenotype development

**Figure 4:**
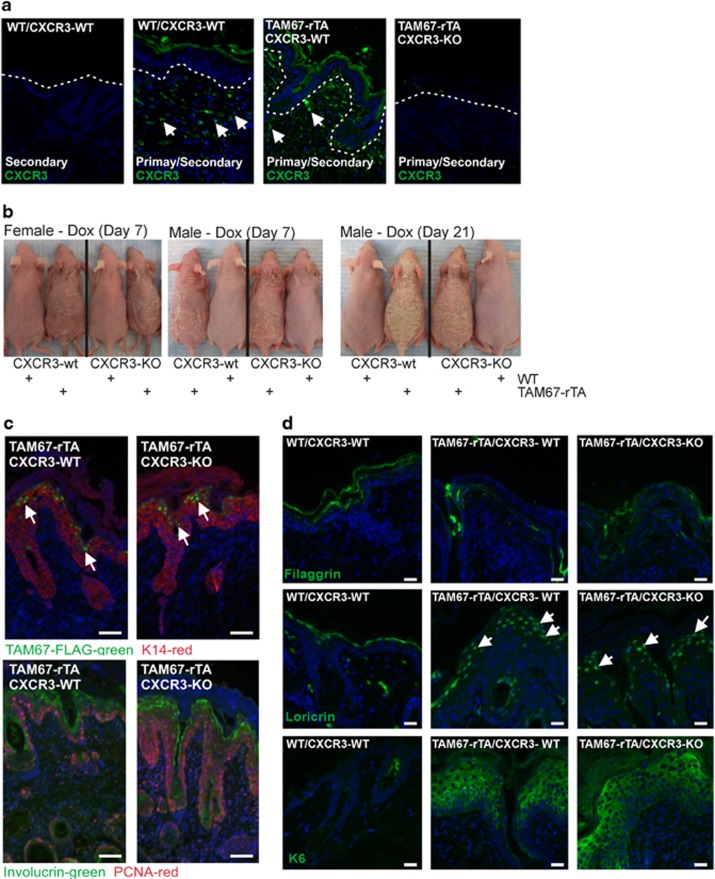
CXCR3 knockout does not prevent development of the keratoderma-like phenotype (**a**) Immunofluorescent staining of CXCR3 in the indicated mouse genotypes. The dotted white line indicates dermal-epidermal junction. Arrows indicated positive CXCR3 staining which is absent in CXCR3-KO mice. (**b**) Mice were treated for 7 and 21 d with 2 mg/ml doxycycline and the TAM67-rTA/CXCR3-WT and TAM67-rTA/CXCR3-KO mice were photographed alongside WT littermates. (**c**) Mice were treated for 8 d with doxycycline and then epidermal sections were stained with the indicated antibodies and images were generated by fluorescence microscopy. The arrows indicate TAM67-FLAG-positive nuclei (green). The bar=200 *μ*m. (**d**) Distribution of filaggrin, loricrin and K6 in epidermal sections prepared from WT/CXCR3-WT, TAM67-rTA/CXCR3-WT and TAM67-rTA/CXCR3-KO mice at 8 d after initiation of doxycycline treatment. The arrows indicate nuclear localization of loricrin in sections from TAM67-expressing mice. The bar=200 *μ*m

CXCL9, CXCL10, and CXCL11 are among the most increased chemokines, as assessed by mRNA and protein level, in TAM67-expressing epidermis (Figure 2). Considering that CXCR3, the receptor for CXCL9, CXCL10 and CXCL11, is expressed by keratinocytes where it can stimulate proliferation^24, 25^ and in activated T-lymphocytes as a chemotaxis mediator,^26^ we tested whether CXCR3 receptor function is required for generation of the TAM67-rTA mouse phenotype. Although CXCR3 ligand (CXCL9, CXCL10, CXCL11) levels are elevated, Figure 4a (and Figure 3b) shows that CXCR3 receptor level is not changed in 8 d wild-type *versus* TAM67-positive epidermis (in the CXCR3-WT background), indicating that altered receptor level is not involved in generating the TAM67-associated epidermal phenotype. To examine the role of these chemokines and receptor, we produced TAM67-rTA/CXCR3-WT and TAM67-rTA/CXCR3-KO mice and monitored the impact on the TAM67-dependent phenotype. We treated WT/CXCR3-WT, TAM67-rTA/CXCR3-WT and TAM67-rTA/CXCR3-KO mice with doxycycline. Immunostaining confirms that CXCR3 is present in WT/CXCR3-WT and TAM67-rTA/CXCR3-WT mice but not in TAM67-rTA/CXCR3-KO mice (Figure 4a). We next monitored the effect of CXCR3 knockout on the TAM67-associated phenotype. Figure 4b shows that the presence or absence of CXCR3 does not alter phenotype severity in male or female mice. A severe flaking/scaly epidermal phenotype develops in the TAM67-expressing mice independent of whether CXCR3 is absent or present.

To get a more detailed view of the impact of CXCR3 knockout on phenotype, we monitored epidermal marker expression as a measure of differentiation status. Figure 4c confirms that TAM67-FLAG is expressed in the nuclei of suprabasal keratinocytes (arrows).^9, 16, 27^ K14, which in normal epidermis marks the epidermal basal layer,^1^ is expressed in all epidermal layers in TAM67-expressing epidermis confirming expansion of the proliferative layer and delayed differentiation.^9^ Multilayer staining of K14 is observed in the TAM67-expressing epidermis in the presence or absence of CXCR3. Involucrin, a suprabasal marker,^28, 29, 30^ is confined to the suprabasal layer in all mice. PCNA staining, an indicator of proliferation, which is confined to the basal layer in normal epidermis, is observed in the basal and suprabasal layers which is indicative of hyperproliferation,^9, 27^ and CXCR3 status does not influence this distribution (Figure 4c). Figure 4d shows reduced filaggrin level, abnormal nuclear accumulation of loricrin (arrows) and induction of expression of hyperproliferation-associated K6 in TAM67-expressing epidermis. These changes are identical in the CXCR3-WT and CXCR3-KO background. Finally, we used a pharmacologic approach to monitor the role of CXCR3, and showed that treatment with a CXCR3-selective inhibitor, AMG487, does not alter phenotype development (not shown). Based on this we conclude that CXCR3 (CXCL9/10/11) signaling is not required for phenotype development.

### Role of S100A8 and S100A9 in phenotype development

**Figure 5:**
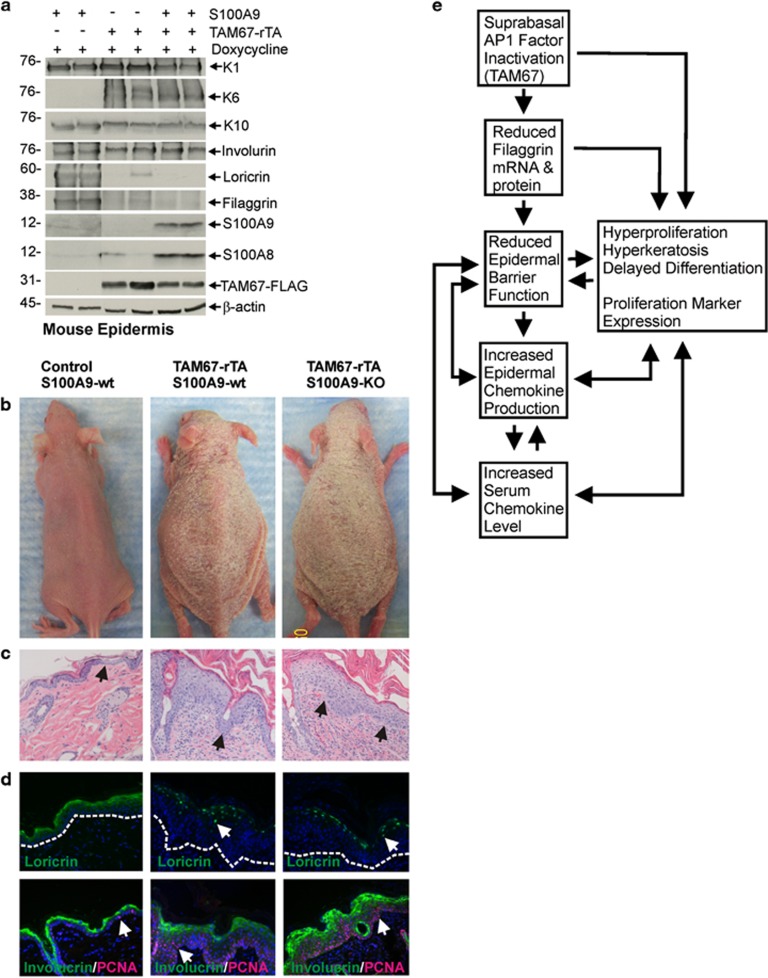
S100A8/A9 knockout does not prevent the keratoderma-like phenotype (**a**) Mice of the indicated TAM67-rTA and S100A9 genotypes were treated for 10 d with doxycycline and epidermal extracts were prepared for immunoblot. (**b**) WT/S100A9-WT, TAM67-rTA/S100A9-WT and TAM67-rTA/S100A9-KO mice were treated for 10 d with doxycycline before image collection. (**c**) H&E stained sections of mouse skin corresponding to mice from panel (**b**). Black arrows indicate the dermal-epidermal junction. (**d**) Loricrin, involucrin, and PCNA staining of epidermis from 10 d doxycycline-treated mice of the indicated genotypes. Dotted white lines indicate the dermal-epidermal junction and the arrows indicate either nuclear loricrin (upper panels) or nuclear PCNA staining (bottom panels). The arrows indicate nuclear loricrin localization (upper panels) and PCNA-positive cells (lower panels). (**e**) Model of phenotype development: AP1 factor inaction leads to a reduction in filaggrin level and also has a direct impact on expression of other genes that control proliferation and differentiation. Loss of filaggrin leads to reduced barrier function,^17, 18^ which exposes the mice to antigens that stimulate epidermal chemokine production and accumulation in the serum which further stimulate phenotype development. The arrows indicate the complex interactive feedback which is likely to exist

Our previous study showed that S100A8 and S100A9 are among the most upregulated genes in TAM67-positive epidermis at both the mRNA and protein level.^16^ We therefore sought to identify a potential role for these calcium-binding, proliferation and inflammation-associated proteins, in phenotype development in the TAM67-expressing epidermis. We bred the TAM67-rTA mice into a S100A9-KO background. S100A9-KO mice are interesting in that S100A9 knockout also results in loss of S100A8. Thus, these mice are deficient in both proteins.^31^ We then characterized various features of phenotype development. Consistent with our previous report,^16^ TAM67-expression is associated with increased expression of K6, S100A8 and S100A9 and reduced levels of loricrin and filaggrin (Figure 5a). As expected, based on our previous reports, the level of K1, K10 and involucrin, which are suprabasal differentiation markers, remain relatively unchanged (Figure 5a). Figures 5b shows the epidermal morphology and histology of control, TAM67-rTA/S100A9-WT and TAM67-rTA/S100A9-KO mice, and shows that phenotype development, as measured by gross appearance and by histology, is not significantly altered in the S100A9-KO environment. The TAM67-positive epidermis displays a scaly appearance with hyperplasia and hyperkeratosis regardless of S100 protein status. Figure 5d confirms nuclear loricrin localization in TAM67 expressing epidermis. Involucrin expression is detected in the suprabasal layers in all mice, and the PCNA proliferation marker, is distributed in both basal and suprabasal epidermal compartments in TAM67-positive mice independent of the presence or absence of S100A8/A9 (Figure 5d).

## Discussion

### Morphological and biochemical response to AP1 factor inactivation

Inhibition of suprabasal AP1 transcription factor function in murine suprabasal epidermis initiates a poorly-understood cascade of changes in epidermal structure and function that produce a profound change in phenotype including nuclear loricrin accumulation, tail and digit autoamputation, hyperproliferation, hyperkeratosis, parakeratosis, delayed differentiation and reduced barrier integrity.^16, 27^ This is coupled with suprabasal expression of hyperproliferation-associated epidermal markers (K6, K14, K16) and reduced expression of filaggrin family members.^16, 27^ Our present findings show that this phenotype includes increased erythroderma and vascular permeabilization. Many of these features are observed in human epidermis in ichthyosis/keratoderma.^17, 18, 20^

These changes may be due to direct effects of AP1 factor loss on gene expression (e.g., filaggrin) and/or indirect effects associated with altered differentiation and reduced barrier function. A key example is the reduction in filaggrin mRNA and protein levels (Figure 5a).^16^ As reviewed by Gutowska and Ogg, a number of chemokines (IL-4, IL-13, TNF*α*, IL-17, IL-22, and IL-24) are reported to suppress filaggrin mRNA and protein level;^32^ however, we do not observe a major increase in these chemokines suggesting this is not a likely explanation for the reduction in filaggrin expression. Alternatively, AP1 transcription factors bind directly to the filaggrin gene promoter to increase gene expression.^33^ Thus, loss of AP1 factor function may directly lead to reduced filaggrin mRNA and filaggrin protein. This finding is consistent with our report showing that the level of filaggrin encoding mRNA is reduced in TAM67-positive epidermis.^16^

### Early chemokine response

We also monitored the impact on AP1 factor inactivation on chemokine production. We observe increased CCL1 and CXCL1 levels in serum at early times (12–24 h) after TAM67 induction. We propose that these chemokines are produced in the epidermis and released to the serum, as we find elevated levels of CCL1 (11-fold) and CXCL1 (2.5-fold) mRNA at 2 d in epidermis. This increase in CCL1 (8-fold) and CXCL1 (2.5-fold) chemokines is maintained in 8 d epidermis. Keratinocyte-produced CCL1 targets CCR8 expressing T-lymphocytes and Langerhans cell precursors, and CXCL1 targets the CXCR2 receptor on keratinocytes^34^ and neutrophils^35^ to promote proliferation and migration. Messenger RNA encoding other chemokines are elevated in epidermis at 2 d including CCL2, CCL3, CCL4, CCL5 (RANTES), CCL7, CCL11, CCL17, CCL20, CXCL9, CXCL10, CXCL11, and IFN*γ*. CCL1 (11-fold) and CCL20 (6-fold) levels are markedly increased. CCL20 is a keratinocyte-produced chemokine that has a role in recruiting CCR6-positive immature dendritic cells and T-lymphocytes from blood.^36, 37, 38^ It is likely that these chemokines play a role in initiating and maintaining erythroderma.

### Intermediate chemokine response

Additional chemokine changes are observed at 8 days after TAM67-induction. These are associated with keratinocyte hyperproliferation and hyperkeratosis, and increased thickening of the epidermis. Analysis of mRNA isolated from 8 d epidermis reveals a marked increase in CCL2 (39-fold), CCL5 (RANTES) (33-fold), CCL7 (80-fold), CCL11 (33-fold) and CXCL10 (over 600-fold) which is associated with accumulation of CCL2, CCL12, CXCL9, CXCL10, IL-16 and IL-IF3 in epidermis and serum (Figure 2). CXCL9 and CXCL10 are the most increased in serum (approximately 40-fold). Many of these chemokines are elevated in states of chronic inflammation and are involved in leukocyte recruitment. For example, CCL2 recruits dendritic and Langerhans cells, CCL5 recruits neutrophils, and CXCL9 and CXCL10 recruit T-lymphocytes. We observed increased accumulation of CD3-positive T-lymphocytes, CD11b-positive neutrophils and F4/80-positive macrophages in the epidermis at 8 d. It is interesting that the CD3-positive T-lymphocytes accumulate in the suprabasal epidermis.

### Late chemokine response

At 21 d the mice manifest an extensive scaling phenotype coupled with hyperproliferation and hyperkeratosis. This is associated with additional changes in the immune response including dermal accumulation of mast cells. Mast cell invasion of the epidermis is associated with response to allergens or pathogens,^39^ which is consistent with the finding that TAM67 expression in embryonic epidermis compromises the barrier. The chemokine profiles remain generally very similar in 21 d mice as compared to 8 d, and the most highly elevated chemokines in the serum include CCL1, CXCL9, and CXCL10. It is interesting that the increase in chemokine level is nearly always associated with a parallel increase in the corresponding mRNA. We are not sure of the mechanism responsible for the changes in chemokine mRNA level. This could be due to gene activation associated with loss of AP1 factor-dependent repression, infiltration of cell types that encode the RNA, or indirect secondary effects (altered differentiation, reduced barrier function).

### Impact of CXCR3 and S100A9 knockout on TAM67-rTA phenotype

The above studies document wide ranging changes in chemokine expression in response to AP1 inactivation. To investigate the role of individual chemokine/chemokine receptors, we focused on CXCL9/10/11 and the CXCR3 receptor, and S100A8 and S100A9. CXCL9, 10 and 11 interact specifically with the CXCR3 receptor,^40, 41^ and are among the most highly elevated chemokines at days 8 and 21 after TAM67 induction. To study their role in phenotype development, we bred the TAM67-rTA mice with CXCR3 knockout mice.^24, 42, 43^ We anticipated that eliminating CXCR3-related signaling may attenuate development of the phenotype associated with AP1 factor deficiency. However, elimination of CXCR3 signaling did not impact phenotype development. The mice develop the same morphological and histological changes including hyperproliferation and hyperkeratosis, and abnormal expression and subcellular distribution of K14, K6, filaggrin and loricrin, suggesting that CXCR3 related ligands are marginally important in phenotype development.

S100A8 and S100A9 are key inflammatory and anti-bacterial proteins in epidermis^44, 45, 46, 47^ that are highly elevated in some epidermal diseases.^45, 48, 49^ Recent studies indicate that S100A9 genetic deletion reduces phenotype severity in mouse inflammatory disease models;^48^ however, we did not observe an appreciable change in disease onset or severity in a S100A9-null/S100A8-null environment. Likewise, S100A8 and S100A9 have been reported to stimulate keratinocyte proliferation which often controls epidermal thickness,^50^ but we did not detect any change in epidermal thickness in the S100A8/A9 null environment.

### AP1 factors in epidermis

AP1 factors are key controllers of differentiation-associated gene expression in epidermis^29, 51, 52, 53, 54^ and loss of AP1 function is associated with striking changes in epidermal differentiation that can mimic disease.^9, 10, 27, 55, 56^ Inactivation of AP1 factor function in the suprabasal epidermis of TAM67-rTA mice results in a phenotype that includes features of ichthyosis vulgaris (altered epidermal differentiation, reduced filaggrin content)^57^ and loricrin keratoderma (pseudoainhum, nuclear loricrin accumulation).^58, 59^ A possible model of phenotype development is present in Figure 5e. In this model, AP1 factor inactivation leads to a reduction in filaggrin level and also has a direct impact on expression of other genes that control proliferation and differentiation. Filaggrin is an essential barrier component that is functionally inactivated in ichthyosis leading to reduced barrier function.^17, 18^ We propose that loss of filaggrin leads to reduced barrier integrity and exposes the mice to antigens that stimulate epidermal chemokine production and accumulation in the serum. Increased chemokine accumulation would also be expected to further influence keratinocyte differentiation status and barrier function. Our present studies describe many chemokine changes, but clearly show, using genetic methods, that S100A8/S100A9 and CXCR3 signaling (CXCL9/10/11) are not required for phenotype development.

## Materials and Methods

### SKH1-TAM67-rTA mice

TAM67 is a dominant-negative form of c-jun which lacks the c-jun amino terminus.^13^ TAM67 forms complexes with all AP1 transcription factors but fails to activate transcription.^13^ This creates an AP1 factor function-deficient environment. We cloned TAM67-FLAG into pTRE-Tight to produce pTRE-Tight-TAM67-FLAG. The Tet*O*-TAM67-FLAG-SV40 transcription cassette from this plasmid was microinjected into B6SJL embryos^9^ to produce TAM67-FLAG transgenic (strain 44) mice and the construct was then bred into an SKH-1 genetic background.^9^ A FLAG epitope is included at the carboxyl terminus of TAM67 to facilitate detection.^9^ We also utilize hINV-rTA transgenic mice, which harbor an expression cassette encoding the hINV promoter linked to rTA, maintained in an SKH-1 genetic background.^60^ TAM67-FLAG and hINV-rTA mice are bred to produce SKH1-TAM67-rTA mice. These mice express rTA in the suprabasal epidermal layers and addition of doxycycline converts rTA to a form that binds to the Tet*O* to turn on TAM67-FLAG expression in the suprabasal epidermis.^9^ The mice are genotyped using DNA-dependent PCR and primers that detect each transgene.^9^ The SKH-1 genetic background was utilized in these studies because the mice are hairless and this facilitates visualization of the epidermis. SKH-1 mice are immune-competent. Mice were maintained in the University Of Maryland School Of Medicine animal facility. The protocols were approved by the Institutional Animal Care and Use Committee and comply with all NIH regulations. Induction of TAM67 expression was achieved by treating animals with 2 mg/ml doxycycline in drinking water. Statistical analysis used the student’s *t*-test.

### CXCR3-KO and S100A9-KO mice

CXCR3 knockout (CXCR3-KO) mice were kindly provided by Dr. Bao Lu from Children’s Hospital, Boston, MA.^24, 42, 43^ S100A9 knockout (S100A9-KO) mice, which lack both S100A8 and S100A9,^31^ were kindly provided by Dr. Donna Kusewitt from MD Anderson Cancer Center, Houston, TX. Both CXCR3-KO and S100A9-KO mice were bred into an SKH-1 hairless genetic background for five generations then bred to the TAM67-FLAG and hINV-rTA mice separately. Then TAM67-FLAG/CXCR3-KO mice were crossed with hINV-rTA/CXCR3-KO mice to yield TAM67-FLAG-rTA/CXCR3-KO mice for experiments. Likewise, TAM67-FLAG/S100A9-KO mice were crossed with hINV-rTA/S100A9-KO mice to yield TAM67-FLAG-rTA/S100A9-KO mice for experiments.

### Antibodies and immunological methods

Immunofluorescence was performed using paraffin-embedded formalin-fixed sections.^9^ K1 (PRB-165 P), K6 (PRB-169 P), K10 (PRB-159 P), K14 (PRB-155 P), filaggrin (PRB-417 P) and loricrin (PRB-145 P) antibodies were from Covance (Emeryville, CA, USA), and *β*-actin (A5441) and FITC conjugated-anti-FLAG (M2) (F4049) antibodies were from (Sigma). CD3 (ab5690) was purchased from Abcam (Cambridge, MA, USA). Antibodies specific for proliferating cell nuclear antigen (PCNA, sc-56) CXCR3 (sc-6226), S100A8 (sc-8113) and S100A9 (sc-8115) were purchased from Santa Cruz (Santa Cruz, CA, USA). Anti-involucrin was prepared in our laboratory. All fluorophore-conjugated secondary antibodies were from Invitrogen (Carlsbad, CA, USA). These included Cy3-conjugated goat anti-rat IGG (A10522) and Alexafluor 488-conjugated goat anti-rabbit IgG (A11031). For immunoblot analysis, epidermis was separated from dermis, frozen in liquid nitrogen, pulverized and suspended in dye-free Laemmli sample buffer.^9^ The suspension was sonicated, centrifuged at 14,000 g, and the soluble extract electrophoresed on a polyacrylamide gel, transferred to nitrocellulose and immunoblotted.^3, 4^ Unless otherwise indicated, immunohistological and immunoblot results were repeated in three separate experiments and sections and extracts were monitored from epidermis of three mice per treatment group. Evans Blue dye (E2129) and toluidine blue (89640) were obtained from Sigma-Aldrich (St. Louis, MO).

### Cytokine mRNA Array

Total RNA was extracted using Illustra RNAspin Mini Isolation Kit (25-0500-70, GE Healthcare), and 0.5 *μ*g of total RNA was reverse transcribed to cDNA using RT^2^ First Strand Kit (330401, QIAGEN, Germantown, MD, USA) according to manufacturers’ protocol. RT^2^ SYBR Green qPCR Mastermix (330500, QIAGEN) was prepared and cDNA added to the mix. PCR component mix (25 *μ*l) was dispensed into each well of the RT^2^ Profiler PCR array (Mouse Cytokines & Chemokines, PAMM-150ZF-6, QIAGEN), sealed, spun down and gene expression was measured by quantitative PCR using Roche LightCycler 480 System. Relative mRNA level was analyzed by the comparative C_T_ method.

### Cytokine protein array

The Mouse Cytokine Array Panel A from R&D Systems (ARY006, Minneapolis, MN, USA) was used to assess epidermal cytokine protein levels. This array has 40 target cytokines including 17 interleukins and 15 chemokines. Briefly, tissue lysates were homogenized in PBS with protease inhibitors and Triton X-100. Sample protein concentrations were quantified and 300 μg protein or 200 μl of serum was added to the prepared membranes. Next the membranes were incubated with the detection antibody cocktail and washed. Streptavidin-HRP and chemiluminescent detection reagents were added sequentially and the membranes were exposed to X-ray film. Pixel densities were quantified using Image J. Each signal doublet was normalized to the reference signal and then to the signal from TAM67-rTA mice not treated with doxycycline.

### AMG487 formulation

AMG487, a CXCR3 inhibitor kindly provided by Amgen (San Francisco, CA, USA) was prepared, as previously described^61^ with modifications as follows. AMG487 was dissolved in dimethyl sulfoxide at 20 mg/ml and then diluted with a 20% solution of 2-hydroxypropyl-*β*-cyclodextrin in water to a final concentration of 1.75 mg/ml. Mice were injected subcutaneously with 0.1 ml to achieve a final AMG487 concentration of 5 mg/kg body weight. Mice received 0.1 ml of either the inhibitor or vehicle injected subcutaneously. The drug and vehicle were stored at 4 °C for the duration of the experiment.

### Flow cytometry

Six to eight week old mice were treated for 2–8 d with 0 or 2 mg/ml doxycycline in drinking water and then back skin was harvested and incubated in 1% trypsin for 1 h at 37 °C. The trypsin was inactivated by addition of 1 volume of 0.5 mg/ml soy bean trypsin inhibitor and the solution was gently pipetted to produce a single-cell suspension. The cells were pelleted and resuspended in calcium-free KSFM, which was supplemented to a final concentration of 0.05 mM calcium chloride and chunks were removed by passage through a sterile nylon strainer (70 *μ*m pore size, BD Falcon 352350). The cells were then resuspended in phosphate-buffered saline and 5–10 million cells were incubated (per sample) with the appropriate antibody for 30 min on ice. The cells were washed twice with PBS and then sorted using a FACSCanto II Sorter. Antibodies for cell sorting include rat anti-mouse CD11b APC (17-0112-81), rat anti-mouse F4/80 APC (17-4801-80), which detects mature macrophages, CD3 APC (17-0032-80), and hamster anti-mouse-CD183 (CXCR3) FITC (11-1831-80), which detects CXCR3, were purchased from Ebioscience (San Diego, CA).

### Blood vessel permeability

A 0.5% sterile solution of Evans Blue was prepared in PBS and filter-sterilized to remove any particulates and 200 *μ*l of the Evans Blue solution was injected into the tail vein. After 30 min the mice were killed by cervical dislocation and photographed. Whole skin biopsies were collected, weighted, and placed into a tube containing 500 *μ*l formamide and incubated for 24–48 h at 55  °C to extract the Evans Blue dye. The tubes were then centrifuged and absorbance of the formamide/Evans Blue mixture was measured at 610 nm using formamide as the blank. Evans Blue uptake was monitored in five mice at each time point.

## References

[bib1] Eckert RL, Crish JF, Robinson NA. The epidermal keratinocyte as a model for the study of gene regulation and cell differentiation. Physiol Rev 1997; 77: 397–424. 9114819 10.1152/physrev.1997.77.2.397

[bib2] Crish JF, Zaim TM, Eckert RL. The distal regulatory region of the human involucrin promoter is required for expression in epidermis. J Biol Chem 1998; 273: 30460–30465. 9804813 10.1074/jbc.273.46.30460

[bib3] Crish JF, Bone F, Banks EB, Eckert RL. The human involucrin gene contains spatially distinct regulatory elements that regulate expression during early versus late epidermal differentiation. Oncogene 2002; 21: 738–747. 11850802 10.1038/sj.onc.1205038

[bib4] Crish JF, Gopalakrishnan R, Bone F, Gilliam AC, Eckert RL. The distal and proximal regulatory regions of the involucrin gene promoter have distinct functions and are required for *in vivo* involucrin expression. J Invest Dermatol 2006; 126: 305–314. 16374477 10.1038/sj.jid.5700019

[bib5] Eckert RL, Efimova T, Balasubramanian S, Crish JF, Bone F, Dashti S. p38 Mitogen-Activated Protein Kinases on the Body Surface - A Function for p38delta. J Invest Dermatol 2003; 120: 823–828. 12713588 10.1046/j.1523-1747.2003.12120.x

[bib6] Efimova T, LaCelle P, Welter JF, Eckert RL. Regulation of human involucrin promoter activity by a protein kinase C, Ras, MEKK1, MEK3, p38/RK, AP1 signal transduction pathway. J Biol Chem 1998; 273: 24387–24395. 9733728 10.1074/jbc.273.38.24387

[bib7] Efimova T, Broome AM, Eckert RL. A regulatory role for p38 delta MAPK in keratinocyte differentiation. Evidence for p38 delta-ERK1/2 complex formation. J Biol Chem 2003; 278: 34277–34285. 12810719 10.1074/jbc.M302759200

[bib8] Angel P, Szabowski A, Schorpp-Kistner M. Function and regulation of AP-1 subunits in skin physiology and pathology. Oncogene 2001; 20: 2413–2423. 11402337 10.1038/sj.onc.1204380

[bib9] Rorke EA, Adhikary G, Jans R, Crish JF, Eckert RL. AP1 factor inactivation in the suprabasal epidermis causes increased epidermal hyperproliferation and hyperkeratosis but reduced carcinogen-dependent tumor formation. Oncogene 2010; 29: 5873–5882. 20818430 10.1038/onc.2010.315 PMC2974027

[bib10] Zenz R, Eferl R, Scheinecker C, Redlich K, Smolen J, Schonthaler HB et al. Activator protein 1 (Fos/Jun) functions in inflammatory bone and skin disease. Arthritis Res Ther 2008; 10: 201. 18226189 10.1186/ar2338 PMC2374460

[bib11] Zenz R, Eferl R, Kenner L, Florin L, Hummerich L, Mehic D et al. Psoriasis-like skin disease and arthritis caused by inducible epidermal deletion of Jun proteins. Nature 2005; 437: 369–375. 16163348 10.1038/nature03963

[bib12] Zenz R, Wagner EF. Jun signalling in the epidermis: from developmental defects to psoriasis and skin tumors. Int J Biochem Cell Biol 2006; 38: 1043–1049. 16423552 10.1016/j.biocel.2005.11.011

[bib13] Brown PH, Chen TK, Birrer MJ. Mechanism of action of a dominant-negative mutant of c-Jun. Oncogene 1994; 9: 791–799. 8108121

[bib14] Thompson EJ, Gupta A, Stratton MS, Bowden GT. Mechanism of action of a dominant negative c-jun mutant in inhibiting activator protein-1 activation. Mol Carcinog 2002; 35: 157–162. 12489106 10.1002/mc.10090

[bib15] Brown PH, Kim SH, Wise SC, Sabichi AL, Birrer MJ. Dominant-negative mutants of cJun inhibit AP-1 activity through multiple mechanisms and with different potencies. Cell Growth Differ 1996; 7: 1013–1021. 8853897

[bib16] Rorke EA, Adhikary G, Young CA, Rice RH, Elias PM, Crumrine D et al. Structural and biochemical changes underlying a keratoderma-like phenotype in mice lacking suprabasal AP1 transcription factor function. Cell Death Dis 2015; 6: e1647. 25695600 10.1038/cddis.2015.21 PMC4669787

[bib17] McLean WH. Filaggrin failure - from ichthyosis vulgaris to atopic eczema and beyond. Br. J Dermatol. 2016; 175(Suppl 2): S4–S7. 10.1111/bjd.14997 PMC5053269 27667308

[bib18] Smith FJ, Irvine AD, Terron-Kwiatkowski A, Sandilands A, Campbell LE, Zhao Y et al. Loss-of-function mutations in the gene encoding filaggrin cause ichthyosis vulgaris. Nat Genet 2006; 38: 337–342. 16444271 10.1038/ng1743

[bib19] Kawasaki H, Kubo A, Sasaki T, Amagai M. Loss-of-function mutations within the filaggrin gene and atopic dermatitis. Curr Probl Dermatol 2011; 41: 35–46. 21576945 10.1159/000323291

[bib20] Kawasaki H, Nagao K, Kubo A, Hata T, Shimizu A, Mizuno H et al. Altered stratum corneum barrier and enhanced percutaneous immune responses in filaggrin-null mice. J Allergy Clin Immunol 2012; 129: 1538–1546. 22409988 10.1016/j.jaci.2012.01.068

[bib21] Nedoszytko B, Sokolowska-Wojdylo M, Ruckemann-Dziurdzinska K, Roszkiewicz J, Nowicki RJ. Chemokines and cytokines network in the pathogenesis of the inflammatory skin diseases: atopic dermatitis, psoriasis and skin mastocytosis. Postepy Dermatol Alergol 2014; 31: 84–91. 25097473 10.5114/pdia.2014.40920 PMC4112246

[bib22] Radu M, Chernoff J. An *in vivo* assay to test blood vessel permeability. J Vis Exp 2013; 73: e50062. 10.3791/50062 PMC3639515 23524912

[bib23] Wang G, Savinko T, Wolff H, Dieu-Nosjean MC, Kemeny L, Homey B et al. Repeated epicutaneous exposures to ovalbumin progressively induce atopic dermatitis-like skin lesions in mice. Clin Exp Allergy 2007; 37: 151–161. 17210053 10.1111/j.1365-2222.2006.02621.x

[bib24] Yates CC, Whaley D, Kulasekeran P, Hancock WW, Lu B, Bodnar R et al. Delayed and deficient dermal maturation in mice lacking the CXCR3 ELR-negative CXC chemokine receptor. Am J Pathol 2007; 171: 484–495. 17600132 10.2353/ajpath.2007.061092 PMC1934531

[bib25] Winkler AE, Brotman JJ, Pittman ME, Judd NP, Lewis JS Jr., Schreiber RD et al. CXCR3 enhances a T-cell-dependent epidermal proliferative response and promotes skin tumorigenesis. Cancer Res 2011; 71: 5707–5716. 21734014 10.1158/0008-5472.CAN-11-0907 PMC3165086

[bib26] Qin S, Rottman JB, Myers P, Kassam N, Weinblatt M, Loetscher M et al. The chemokine receptors CXCR3 and CCR5 mark subsets of T cells associated with certain inflammatory reactions. J Clin Invest 1998; 101: 746–754. 9466968 10.1172/JCI1422 PMC508621

[bib27] Rorke EA, Adhikary G, Young CA, Roop DR, Eckert RL. Suppressing AP1 factor signaling in the suprabasal epidermis produces a keratoderma phenotype. J Invest Dermatol 2015; 135: 170–180. 25050598 10.1038/jid.2014.310 PMC4268309

[bib28] Eckert RL, Green H. Structure and evolution of the human involucrin gene. Cell 1986; 46: 583–589. 2873896 10.1016/0092-8674(86)90884-6

[bib29] Eckert RL, Crish JF, Efimova T, Dashti SR, Deucher A, Bone F et al. Regulation of involucrin gene expression. J Invest Dermatol 2004; 123: 13–22. 15191537 10.1111/j.0022-202X.2004.22723.x

[bib30] Rice RH, Green H. Presence in human epidermal cells of a soluble protein precursor of the cross-linked envelope: activation of the cross-linking by calcium ions. Cell 1979; 18: 681–694. 42494 10.1016/0092-8674(79)90123-5

[bib31] Manitz MP, Horst B, Seeliger S, Strey A, Skryabin BV, Gunzer M et al. Loss of S100A9 (MRP14) results in reduced interleukin-8-induced CD11b surface expression, a polarized microfilament system, and diminished responsiveness to chemoattractants *in vitro*. Mol Cell Biol 2003; 23: 1034–1043. 12529407 10.1128/MCB.23.3.1034-1043.2003 PMC140712

[bib32] Gutowska-Owsiak D, Ogg GS. Cytokine regulation of the epidermal barrier. Clin Exp Allergy 2013; 43: 586–598. 23711120 10.1111/cea.12023

[bib33] Jang SI, Steinert PM, Markova NG. Activator protein 1 activity is involved in the regulation of the cell type-specific expression from the proximal promoter of the human profilaggrin gene. J Biol Chem 1996; 271: 24105–24114. 8798649 10.1074/jbc.271.39.24105

[bib34] Kulke R, Bornscheuer E, Schluter C, Bartels J, Rowert J, Sticherling M et al. The CXC receptor 2 is overexpressed in psoriatic epidermis. J Invest Dermatol 1998; 110: 90–94. 9424095 10.1046/j.1523-1747.1998.00074.x

[bib35] Cataisson C, Pearson AJ, Tsien MZ, Mascia F, Gao JL, Pastore S et al. CXCR2 ligands and G-CSF mediate PKCalpha-induced intraepidermal inflammation. J Clin Invest 2006; 116: 2757–2766. 16964312 10.1172/JCI27514 PMC1560349

[bib36] Le BM, Etchart N, Goubier A, Lira SA, Sirard JC, van RN et al. Dendritic cells rapidly recruited into epithelial tissues via CCR6/CCL20 are responsible for CD8+ T cell crosspriming *in vivo*. Immunity 2006; 24: 191–201. 16473831 10.1016/j.immuni.2006.01.005

[bib37] Varona R, Cadenas V, Gomez L, Martinez A, Marquez G. CCR6 regulates CD4+ T-cell-mediated acute graft-versus-host disease responses. Blood 2005; 106: 18–26. 15774622 10.1182/blood-2004-08-2996

[bib38] Paradis TJ, Cole SH, Nelson RT, Gladue RP. Essential role of CCR6 in directing activated T cells to the skin during contact hypersensitivity. J Invest Dermatol 2008; 128: 628–633. 17882271 10.1038/sj.jid.5701055

[bib39] Sehra S, Serezani AP, Ocana JA, Travers JB, Kaplan MH. Mast cells regulate epidermal barrier function and the development of allergic skin inflammation. J Invest Dermatol 2016; 136: 1429–1437. 27021404 10.1016/j.jid.2016.03.019 PMC4921316

[bib40] Lacotte S, Brun S, Muller S, Dumortier H. CXCR3, inflammation, and autoimmune diseases. Ann N Y Acad Sci 2009; 1173: 310–317. 19758167 10.1111/j.1749-6632.2009.04813.x

[bib41] Groom JR, Luster AD. CXCR3 ligands: redundant, collaborative and antagonistic functions. Immunol. Cell Biol 2011; 89: 207–215. 21221121 10.1038/icb.2010.158 PMC3863330

[bib42] Hancock WW, Lu B, Gao W, Csizmadia V, Faia K, King JA et al. Requirement of the chemokine receptor CXCR3 for acute allograft rejection. J Exp Med 2000; 192: 1515–1520. 11085753 10.1084/jem.192.10.1515 PMC2193193

[bib43] Yates CC, Krishna P, Whaley D, Bodnar R, Turner T, Wells A. Lack of CXC chemokine receptor 3 signaling leads to hypertrophic and hypercellular scarring. Am J Pathol 2010; 176: 1743–1755. 20203286 10.2353/ajpath.2010.090564 PMC2843466

[bib44] Broome AM, Ryan D, Eckert RL. S100 protein subcellular localization during epidermal differentiation and psoriasis. J Histochem Cytochem 2003; 51: 675–685. 12704215 10.1177/002215540305100513 PMC3785113

[bib45] Eckert RL, Broome AM, Ruse M, Robinson N, Ryan D, Lee K. S100 proteins in the epidermis. J Invest Dermatol 2004; 123: 23–33. 15191538 10.1111/j.0022-202X.2004.22719.x

[bib46] Lee KC, Eckert RL. S100A7 (Psoriasin)–mechanism of antibacterial action in wounds. J Invest Dermatol 2007; 127: 945–957. 17159909 10.1038/sj.jid.5700663

[bib47] Robinson NA, Lapic S, Welter JF, Eckert RL. S100A11, S100A10, annexin I, desmosomal proteins, small proline-rich proteins, plasminogen activator inhibitor-2, and involucrin are components of the cornified envelope of cultured human epidermal keratinocytes. J Biol Chem 1997; 272: 12035–12046. 9115270 10.1074/jbc.272.18.12035

[bib48] Schonthaler HB, Guinea-Viniegra J, Wculek SK, Ruppen I, Ximenez-Embun P, Guio-Carrion A et al. S100A8-S100A9 protein complex mediates psoriasis by regulating the expression of complement factor C3. Immunity 2013; 39: 1171–1181. 24332034 10.1016/j.immuni.2013.11.011

[bib49] Nakajima K, Terao M, Takaishi M, Kataoka S, Goto-Inoue N, Setou M et al. Barrier abnormality due to ceramide deficiency leads to psoriasiform inflammation in a mousemodel. J Invest Dermatol. 2013; 133: 2555–2565. 23633022 10.1038/jid.2013.199

[bib50] Iotzova-Weiss G, Dziunycz PJ, Freiberger SN, Lauchli S, Hafner J, Vogl T et al. S100A8/A9 stimulates keratinocyte proliferation in the development of squamous cell carcinoma of the skin via the receptor for advanced glycation-end products. PLoS ONE 2015; 10: e0120971. 25811984 10.1371/journal.pone.0120971 PMC4374726

[bib51] Eckert RL, Welter JF. Epidermal keratinoctyes–genes and their regulation. Cell Death Differ 1996; 3: 373–383. 17180107

[bib52] Eckert RL, Crish JF, Banks EB, Welter JF. The epidermis: genes on - genes off. J Invest. Dermatol 1997; 109: 501–509. 9326381 10.1111/1523-1747.ep12336477

[bib53] Rossi A, Jang SI, Ceci R, Steinert PM, Markova NG. Effect of AP1 transcription factors on the regulation of transcription in normal human epidermal keratinocytes. J Invest Dermatol 1998; 110: 34–40. 9424084 10.1046/j.1523-1747.1998.00071.x

[bib54] Welter JF, Eckert RL. Differential expression of fos and jun family members c-fos, fosB, Fra-1, Fra-2, c-jun, junB and junD during human epidermal keratinocyte differentiation. Oncogene 1995; 11: 2681–2687. 8545126

[bib55] Mehic D, Bakiri L, Ghannadan M, Wagner EF, Tschachler E. Fos and jun proteins are specifically expressed during differentiation of human keratinocytes. J Invest Dermatol 2005; 124: 212–220. 15654976 10.1111/j.0022-202X.2004.23558.x

[bib56] Zenz R, Wagner EF. Jun signalling in the epidermis: From developmental defects to psoriasis and skin tumors. Int J Biochem Cell Biol 2006; 38: 1043–1049. 16423552 10.1016/j.biocel.2005.11.011

[bib57] Presland RB, Boggess D, Lewis SP, Hull C, Fleckman P, Sundberg JP. Loss of normal profilaggrin and filaggrin in flaky tail (ft/ft) mice: an animal model for the filaggrin-deficient skin disease ichthyosis vulgaris. J Invest Dermatol 2000; 115: 1072–1081. 11121144 10.1046/j.1523-1747.2000.00178.x

[bib58] Matsumoto K, Muto M, Seki S, Saida T, Horiuchi N, Takahashi H et al. Loricrin keratoderma: a cause of congenital ichthyosiform erythroderma and collodion baby. Br J Dermatol 2001; 145: 657–660. 11703298 10.1046/j.1365-2133.2001.04412.x

[bib59] Yeh JM, Yang MH, Chao SC. Collodion baby and loricrin keratoderma: a case report and mutation analysis. Clin Exp Dermatol 2013; 38: 147–150. 22831754 10.1111/j.1365-2230.2012.04426.x

[bib60] Jaubert J, Patel S, Cheng J, Segre JA. Tetracycline-regulated transactivators driven by the involucrin promoter to achieve epidermal conditional gene expression. J Invest Dermatol 2004; 123: 313–318. 15245431 10.1111/j.0022-202X.2004.23203.x

[bib61] Walser TC, Rifat S, Ma X, Kundu N, Ward C, Goloubeva O et al. Antagonism of CXCR3 inhibits lung metastasis in a murine model of metastatic breast cancer. Cancer Res 2006; 66: 7701–7707. 16885372 10.1158/0008-5472.CAN-06-0709

